# Evaluation of the biodegradation of Alaska North Slope oil in microcosms using the biodegradation model BIOB

**DOI:** 10.3389/fmicb.2014.00212

**Published:** 2014-05-14

**Authors:** Jagadish Torlapati, Michel C. Boufadel

**Affiliations:** ^1^Center for Natural Resources Development and Protection, New Jersey Institute of TechnologyNewark, NJ, USA; ^2^Department of Civil and Environmental Engineering, Center for Natural Resources Development and Protection, New Jersey Institute of TechnologyNewark, NJ, USA

**Keywords:** oil spill, biodegradation, numerical modeling

## Abstract

We present the details of a numerical model, BIOB that is capable of simulating the biodegradation of oil entrapped in the sediment. The model uses Monod kinetics to simulate the growth of bacteria in the presence of nutrients and the subsequent consumption of hydrocarbons. The model was used to simulate experimental results of Exxon Valdez oil biodegradation in laboratory columns (Venosa et al., 2010). In that study, samples were collected from three different islands: Eleanor Island (EL107), Knight Island (KN114A), and Smith Island (SM006B), and placed in laboratory microcosms for a duration of 168 days to investigate oil bioremediation through natural attenuation and nutrient amendment. The kinetic parameters of the BIOB model were estimated by fitting to the experimental data using a parameter estimation tool based on Genetic Algorithms (GA). The parameter values of EL107 and KN114A were similar whereas those of SM006B were different from the two other sites; in particular biomass growth at SM006B was four times slower than at the other two islands. Grain size analysis from each site revealed that the specific surface area per unit mass of sediment was considerably lower at SM006B, which suggest that the surface area of sediments is a key control parameter for microbial growth in sediments. Comparison of the BIOB results with exponential decay curves fitted to the data indicated that BIOB provided better fit for KN114A and SM006B in nutrient amended treatments, and for EL107 and KN114A in natural attenuation. In particular, BIOB was able to capture the initial slow biodegradation due to the lag phase in microbial growth. Sensitivity analyses revealed that oil biodegradation at all three locations were sensitive to nutrient concentration whereas SM006B was sensitive to initial biomass concentration due to its slow growth rate. Analyses were also performed to compare the half-lives of individual compounds with that of the overall polycyclic aromatic hydrocarbons (PAHs).

## Introduction

The Exxon Valdez oil spill (EVOS) of 1989 in Prince William Sound (PWS) resulted in the contamination of approximately 2000 km of shoreline within the Gulf of Alaska (Bragg et al., 1994). The more recent Deepwater Horizon oil spill contaminated over 1600 km of shoreline within the Gulf of Mexico (Barron, 2012; Lubchenco et al., 2012). Removal of oil spilled in the environment requires approaches that are both effective and environmentally safe, and one of the techniques is bioremediation, which relies on augmenting the natural biodegradation rate of oil (Atlas, 1995; Vogel, 1996; Xu and Obbard, 2004; Cunliffe and Kertesz, 2006; Karamalidis et al., 2010).

Bioremediation can be studied by conducting laboratory scale experiments (Boufadel et al., 1999; Sabaté et al., 2004; Chen et al., 2008; Tian et al., 2008; Beolchini et al., 2010; Lors et al., 2012) or field scale experiments. Boufadel et al. (1999) evaluated the nitrate concentration required for maximum biodegradation of n-heptadecane in sand columns with monitored oxygen consumption and carbon dioxide production. They reported that the maximum biodegradation occurs at a concentration of 2.5 mg nitrate-N/L. They also speculated nitrogen recycling by biomass when the influent nitrate concentration was zero. This occurred by lysing (break apart) of cells to provide nutrients that other cells could use for growth and biodegradation. Wrenn et al. (2006) studied the effects of nutrient source and supply in crude oil biodegradation. They observed that the extent and rate of oil biodegradation remain unchanged regardless of whether the nutrient supply was intermittent or continuous. Desai et al. (2008) investigated the biodegradation kinetics of individual components and PAHs and their studies indicated that the prediction of natural or enhanced biodegradation of PAHs cannot be based on single compound kinetics as this assumption would overestimate the rate of disappearance, and does not account for the inhibition due to competitive interactions between the compounds.

Venosa et al. (2010) conducted laboratory experiments to study the biodegradation of 19 year old lingering weathered oil in PWS due to EVOS at three different islands. They observed that the most weathered oil was the most biodegradable. The experimental results obtained from laboratory scale experiments could be used to gain a better understanding of the kinetic processes involved in biodegradation by the use of numerical models.

Numerical models have been routinely used by researchers to predict the results. Essaid et al. (2003) used the US Geological Survey (USGS) solute transport and biodegradation code BIOMOC coupled with the USGS universal inverse modeling code UCODE to quantify the BTEX dissolution and biodegradation at a site located in Bemidji, MN. Vilcáez et al. (2013) developed a numerical model for the biodegradation of oil droplets as opposed to dissolved oil, and it was applied to estimate the time scale of biodegradation of Deepwater Horizon oil spill in Gulf of Mexico. The model revealed that small oil droplets biodegraded faster due to their larger surface area per unit mass. Herold et al. (2011) modeled the enhanced bioremediation of groundwater contaminated by acenaphthene, methylbenzofurans, and dimethylbenzofurans. The calibrated model was used to explore the feasibility and efficiency of remediation scenarios. It was observed from their simulations that even with several simplifications made in conceptualization, they were able to demonstrate the ability of their model to detect key processes needed for an effective remediation scheme. Geng et al. (2013) developed a mathematical model to simulate the biodegradation of residual hydrocarbon in a variably-saturated sand column. They estimated the biodegradation kinetic parameters by fitting the model to experimental data of oxygen, CO_2_ and residual mass of heptadecane obtained from two columns. They were also able to predict accurately the biodegradation for three other columns using the same parameters.

The goal of this study is to present a numerical model, BIOB which is capable of simulating the biodegradation of oil entrapped in the sediments, and to estimate its parameters using the experimental results from Venosa et al. (2010). The estimated parameters will be used to predict biodegradation under different environmental conditions. A parameter estimation tool based on Genetic Algorithm (GA) is employed to automatically conducting the fitting. Finally, the sensitivity of the model prediction to the kinetic parameters of the BIOB model is evaluated.

## Biodegradation of Exxon Valdez oil in microcosms

Venosa et al. (2010) conducted laboratory experiments to test the biodegradability of 19 year old lingering oil due to the 1989 EVOS in Alaska, USA. They assessed whether or not oil weathered more than 70% could undergo further biodegradation. Oil weathering could occur as a result of its physical (e.g., evaporation, dissolution, washout), chemical (e.g., photooxidation), or microbial (e.g., biodegradation) processes. Oil weathering by biodegradation can be distinguished from the other processes by using biomarkers (e.g., hopane) to normalize the measured oil concentration. Biomarkers are compounds that biodegrade very slowly within the time frame of interest and have been used to evaluate the effectiveness of biodegradation (Bragg et al., 1994; Venosa et al., 1996). In Venosa et al. (2010), the PAH concentrations were normalized using hopane. The degree of weathering is calculated by comparing the constitution of the weathered oil with the fresh oil. Samples were collected from three different sites of Eleanor Island (EL107), Knight Island (KN114A), and Smith Island (SM006B) whose respective mass weathering indices (MWI) were 30, 76, and 60%. These samples of oil-contaminated sediment were collected from each site by digging up to 10–40 cm below the surface. The volume of each sample was about 50 L and the collected samples were used for microcosm experiments at the University of Cincinnati. Seawater samples from each island were also collected for natural attenuation experiments.

The collected samples were then used for experiments to study (a) natural attenuation, the biodegradation due to the nutrients naturally present in seawater/sediment and (b) nutrient-amended treatment, where nutrients were added to the samples and the concentrations of the nutrients were maintained by adding 10 mg/L of KNO_3_ to ensure no nutrient limitation occurs (Venosa et al., 1996; Boufadel et al., 1999; Du et al., 1999). Each microcosm contained 800 mL volume of mixed sediment weighing approximately 1.5 kg with glass beads of diameter 2 and 3 mm at the top and bottom of the sediment to prevent sediment fines from exiting the unit. A peristaltic pump was used to withdraw seawater from the bottom of the microcosms at a flow rate of 2 mL/min to an overhead reservoir. The overhead reservoir worked by a siphon mechanism and the seawater is released to the natural attenuation microcosms every 4 h. Samples were collected at 0, 14, 28, 56, 112, and 168 days and the temperature during the experimental study was similar to the conditions in PWS which was 15°C.

The sediments from each microcosm were analyzed for hydrocarbons using gas chromatography/mass spectrometry (GC/MS). The Total Kjeldahl Nitrogen (TKN) was measured using the Hach method (Hach et al., 1985). The results obtained from these experimental analyses indicated that there was significant biodegradation in the nutrient-amended samples, even when the MWI was high. The experimental results were fitted by Venosa et al. (2010) using a first-order decay model. Their model showed that the highest biodegradation rate occurred in the most weathered oil (KN114A). The experimental results also showed that natural attenuation (non-nutrient amended microcosms) resulted in significant oil biodegradation although the biodegradation rates were lower compared to the nutrient-amended microcosms. This shows that supplying nutrients to the contaminated sediment increased the biodegradation potential in the microcosms and thereby this could be used to significantly increase the biodegradation rates of PAHs at the contaminated sites.

## Methods

### Numerical model

The numerical model, BIOB, uses Monod kinetics to simulate the biodegradation of hydrocarbon entrapped between the sediments. It also simulates the growth of bacterial biomass in the presence of nutrients. The decay of hydrocarbon can be mathematically expressed as follows (Geng et al., 2014):

(1) $$\frac{dS}{dt}=-\frac{\mu }{Y_{X}}X$$

Where *S* is the concentration of the polycyclic aromatic hydrocarbons (PAHs) (mg S/kg sediment), *X* is the concentration of the biomass (mg X/kg sediment), *Y_x_* is the biomass yield coefficient for growth on the hydrocarbon (mg X/mg S) and μ is the growth rate of the biomass (day^−1^) given by Geng et al. (2014):

(2) $$\mu =\mu_{\max}\left(1-\frac{X}{X_{\max}}\right)\frac{S}{K_{S}+S}\frac{N}{K_{N}+N}$$

Where μ_max_ is the maximum growth rate (day^−1^), *X*_max_ is the maximum allowable microbial concentration (mg X/kg sediment), *K_s_* is the half saturation concentration of PAH (mg S/kg sediment), *N* is the nitrogen-based nutrient concentration (nitrate+nitrite+ammonia) (mg-N/L of pore water), and *K*_*N*_ is the half-saturation concentration for nitrogen consumption (mg-N/L of pore water). The term $\left(1-\frac{X}{X_{\max}}\right)$ was introduced by Geng et al. (2014) to account for the decrease in biomass accumulation when the microbial concentration approaches its maximum value. Geng et al. (2014) also included an expression for hopane removal due to physical processes in Equation 1. However, it was noted in Venosa et al. (2010) study that hopane concentration remained constant throughout the experiment and hence this term was made equal to zero for the purpose of this study. This suggests that the hydrocarbon decay in the microcosms occurred due to the biodegradation process and not due to abiotic processes such as leaching or washout. BIOB also includes equations to solve for the consumption of nutrients, O_2_ and the production of CO_2_ levels. Typically, O_2_ consumption and CO_2_ production could be used to evaluate the microbial growth. Experimentally, the microbial concentration can be estimated using MPN (most probable number) (Alexander, 1965) or qPCR (quantitative polymerase chain reaction) which is used to quantify a specific targeted DNA (Heid et al., 1996; Smith and Osborn, 2009). In the Venosa et al. (2010) study, the nutrients, O_2_ and CO_2_ levels were not measured in the experiments and they were always present in excess, and hence these equations were not considered in the current model. The complete set of equations used in the BIOB model is presented in Supplementary Material (Appendix A1).

The biomass growth can be expressed mathematically as follows (Geng et al., 2014):

(3) $$\frac{dX}{dt}=\left(\mu -k_{d}\right)X$$

Where *k_d_* is the endogenous biomass decay rate (day^−1^).

The model can be solved for the concentration of biomass and PAH at different times by numerically solving the Equations (1) through (3) using an ordinary differential equation (ODE) solver. BIOB uses a Runge–Kutta Felhberg adaptive time-stepping scheme to solve the equations (Chapra and Canale, 1998).

### Inverse problem—parameter estimation

The Monod kinetic model requires several parameters that define the system. Therefore, we need to estimate these model parameters before it were modeled using BIOB. Since no microbial concentration was presented in the study, the maximum microbial concentration (*X*_max_) and the initial microbial concentration (*X*) were also estimated. Therefore, a total of seven parameters were estimated using a parameter estimation tool based on a mathematical tool known as GA (Torlapati, 2013). It should be noted that the GA is only used for estimating the different parameters that are being used in BIOB (Equations 1–3). Table 1 shows the seven parameters that were estimated along with their higher and lower bounds. The general procedure of a GA is described below.

**Table 1:** **Parameters estimated by the model using individual datasets**.

**Parameter**	**Units**	**Lower bound**	**Upper bound**	**Estimated value (EL107)**	**Estimated value (KN114A)**	**Estimated value (SM006B)**	**Literature values**
*X*_max_	mg X/kg sediment	0	2	0.34	1.27	1.04	0.63 (Geng et al., 2014)
*X*_0_	mg X/kg sediment	0	0.5	1.4E-03	3.00E-3	2.46E-3	4.8E-7–4.8E-3 (Köpke et al., 2005)
μ_max_	day^−1^	0.01	10	3.41	3.34	1.35	1.8–9.6 (Nicol et al., 1994)
*k*_*d*_	day^−1^	0.05	0.5	0.38	0.28	0.29	0.05–0.76 (Essaid et al., 1995)
*Y*_*X*_	mg X/mg S	0.001	4	0.28	0.27	0.19	0.01–1.33 (Essaid et al., 1995)
*K*_*S*_	mg S/mg hopane	0.5	500	51.8	43.8	40.2	
*K*_*N*_	mg of N/L of solution	0.02	5	1.56	1.55	1.77	0.1 (Essaid and Bekins, 1997)

*X_0_ is the initial biomass concentration*.

The six key steps involved in a traditional GA are: encoding, population generation, selection, crossover, mutation, and termination (Holland, 1975). The GA starts with a randomly-generated initial set of solutions (also known as chromosomes) between the higher and lower bounds set by the user and this is called the initial population. A solution is a randomly generated parameter between the higher and lower bound set by the user that will used to calculate the objective function. These randomly generated solutions or chromosomes are used to calculate the fitness of the population using the objective function. The objective function value is assigned as the fitness for each chromosome and it is used to assess its ability to survive the current generation. For a minimization problem, a lower value of fitness is desirable. Based on this fitness value, two parents are selected using a selection process. The selected parents undergo a crossover, where the genetic information is exchanged between the parents using a crossover function. Since the genetic information is transferred to the subsequent generation of children, it is always preferable to choose individuals with better fitness in the selection process. It is also possible that an offspring generated from the crossover of the parents could undergo a mutation operation governed by a mutation probability. The fitness of the offspring is calculated and is combined with the entire population. The individuals with poor fitness are removed from the population (death) at the end of the generation. There are several strategies available for the discarding bad solutions, and for implementing the process of encoding, selection, crossover, and mutation. Further details about the GA are available in Torlapati (2013). The specific methods used in this study are discussed in section Results.

### Parameter sensitivity

The sensitivity of the model output to the parameter estimates were evaluated using the following approach. The covariance matrix, *V*_*x*_, of the parameters was evaluated according to the equation (Bard, 1974):

(4) $$V_{x}=\sigma^{2}H^{-1}$$

Where *H* is the Hessian matrix whose terms are the second derivative of the objective function with respect to the parameters. The variance of errors, σ^2^, can be estimated by Bard (1974):

(5) $$\sigma^{2}=\frac{F}{n-p}$$

Where *F* is the value of the objective function at the optimum, *n* is the number of observations and *p* is number of estimated parameters.

## Results

Bioavailability of oil to the degrading microbial communities is an important phenomenon since the biodegradation occurs at the oil-water interface for hydrocarbons with low solubility (Johnsen et al., 2005). Since experimental data was used to calibrate the parameters for the model, the rate of biodegradation for oil with low bioavailability will be lower compared to a location with higher bioavailability. The experimental dataset from the nutrient amended experiments was used to estimate the kinetic parameters required for BIOB. For the inverse problem, an initial population size of 48 was used. This means that 48 random solutions were generated for each parameter between the higher and lower bound given in Table 1. This is to ensure that the estimated parameters do not exceed values observed in the literature. The initial population size determines the solution space in which the GA searches for the parameters. A low initial population might not provide a wide range of solutions and we might be stuck in a local minimum whereas a higher population increases the computational time. It was observed from our simulations that the results did not improve beyond the population size of 48 and hence a population size of 48 was used for all parameter estimation simulations. The number of generations used in our parameter estimation model was 300. The initial hopane-normalized PAH concentrations were (Venosa et al., 2010) 72.65, 44.68, and 58.68 mg/mg hopane for EL107, KN114A, and SM006B, respectively. The concentration of nitrogen (N) in Equation 2 was set to 10 mg/L for the complete duration of 168 days since Venosa et al. (2010) study ensured that there was sufficient nutrient concentration available for nutrient-amended experiments. The input data for the parameter estimation model was experimental data obtained from the study. Since the experiments were performed in triplicates, the average value was obtained at each sample measurement and was provided as an input to the GA. The “fitness” of each solution was then evaluated by calculating the error (difference) between the model concentration and the observed (input) experimental concentration that was provided. This error was normalized by the observed concentration at the corresponding time, and the normalized error was squared, added for all the experimental data, and assigned as fitness for that population. The objective of the GA was to minimize this weighted least squares (WLS) by genetic recombination of the solutions generated in the initial population. This was continued for 300 generations and the solution with the best fitness at the end of the simulation was used for the final parameters. All the simulations were performed on Intel Core i7 desktop computer with 8 cores and a total clock speed of 3.40 GHz. The time step used for all the simulations was 3 h.

Initially, the parameter estimation tool was run with the nutrient amended experimental data for all three islands provided as simultaneous input. The estimated parameters over-predicted the biodegradation for the SM006B dataset. Therefore, the parameters were estimated for each island dataset separately, and the fit improved greatly. The parameters estimated for individual island datasets are presented in Table 1. The estimated parameters that were obtained for each island dataset were compared against each other and it was observed that the EL107 and KN114A datasets had similar parameters. Hence, the parameter estimation tool was run again with the experimental data from the both the islands provided as input to obtain the common solution for these two datasets. The estimated parameters are provided in Table 2 along with the parameters estimated for SM006B. Note that the best fit parameters presented in Table 2 also include the standard deviation for each parameter and the details of these calculations are presented in section Sensitivity Analysis. Subsequently, the best-fit parameters obtained for the nutrient amended experiments (Table 2) were used to estimate the nutrient concentration in the natural attenuation experiments. The natural attenuation microcosms were supplied with seawater collected from each site every 4 h to provide reaeration of the seawater and gentle mixing. The purpose of the parameter estimation tool was to estimate the naturally present nutrients (N) in the seawater that were utilized for biodegradation. The concentrations of nitrogen for natural attenuation estimated by the parameter estimation tool were 0.56, 0.79, and 2.21 mg/L for EL107, KN114A, and SM006B, respectively. The concentration of nutrients in SM006B location was about three to four times higher than the other two locations.

**Table 2:** **Parameters estimated in the model and their best-fit value**.

**Parameter**	**Units**	**Lower bound**	**Upper bound**	**Estimated value (EL107 and KN114A)**	**Estimated value (SM006B)**	**Literature values**
*X*_max_	mg X/kg sediment	0	2	0.40 ± 0.0601^a^	1.04 ± 0.033	0.63 (Geng et al., 2014)
				1.18 ± 0.0601^b^		
*X*_0_	mg X/kg sediment	0	0.5	1.45E-03 ± 1.87^a^	2.46E-03 ± 0.76	4.8E-7–4.8E-3 (Köpke et al., 2005)
				4.34E-03 ± 1.87^b^		
μ_max_	day^−1^	0.01	10	5.12 ± 0.063	1.35 ± 0.013	1.6–6.4 (Nicol et al., 1994)
*k_d_*	day^−1^	0.05	0.5	0.32 ± 0.072	0.29 ± 0.018	0.05–0.76 (Essaid et al., 1995)
*Y_X_*	mg X/mg S	0.001	4	0.28 ± 0.058	0.19 ± 0.029	0.01–1.33 (Essaid et al., 1995)
*K_S_*	mg S/mg hopane	0.5	500	56.9 ± 0.076	40.2 ± 0.023	
*K_N_*	mg of N/L of solution	0.02	5	1.63 ± 0.48	1.77 ± 0.094	0.1 (Essaid and Bekins, 1997)

a *EL107*.

b *KN114A*.

*X_0_ is the initial biomass concentration*.

**Figure 1:**
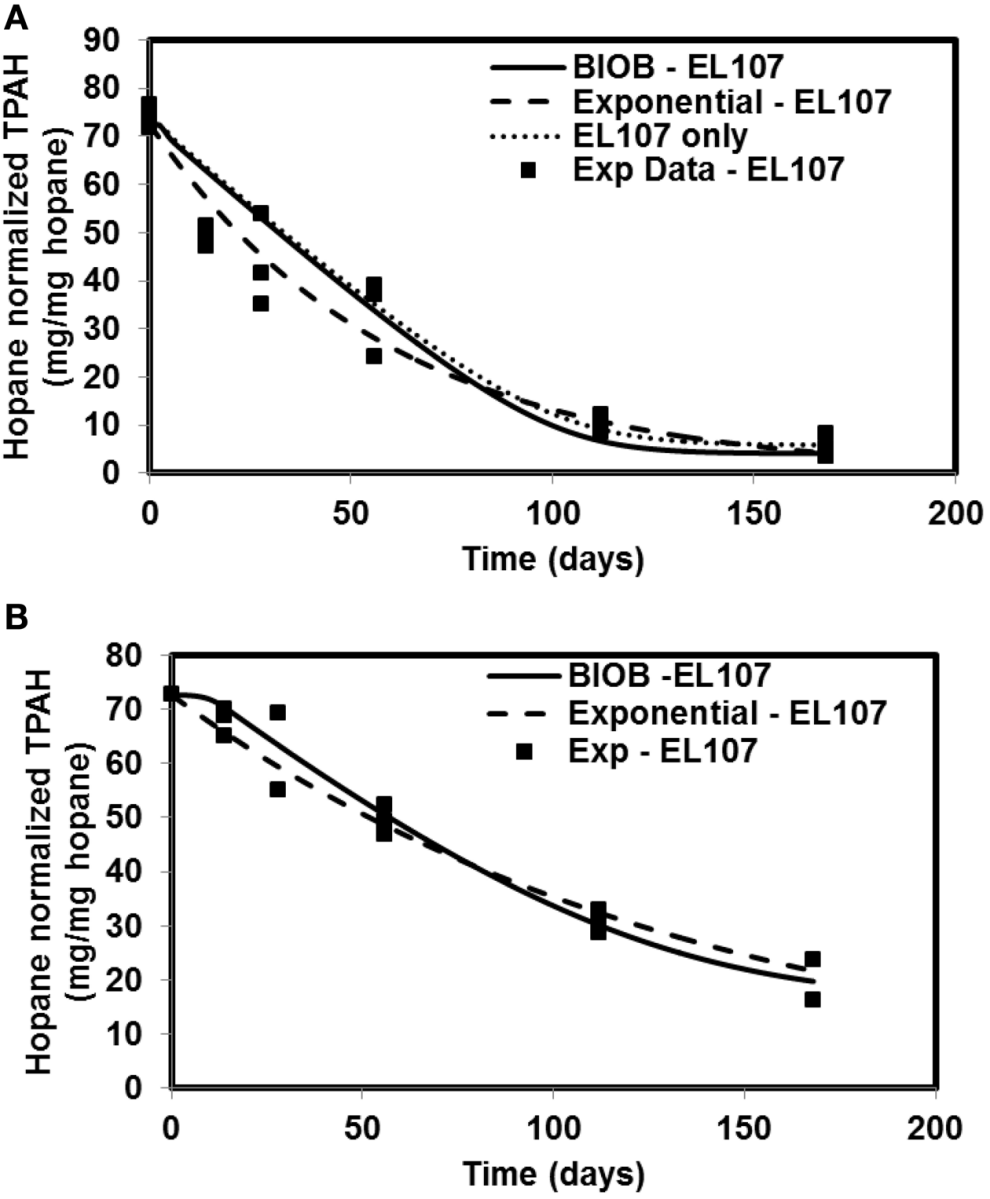
**Comparison of different model results and the experimental results for Eleanor Island (EL107) (A) Nutrient amended experiments (B) Natural attenuation**.

**Figure 2:**
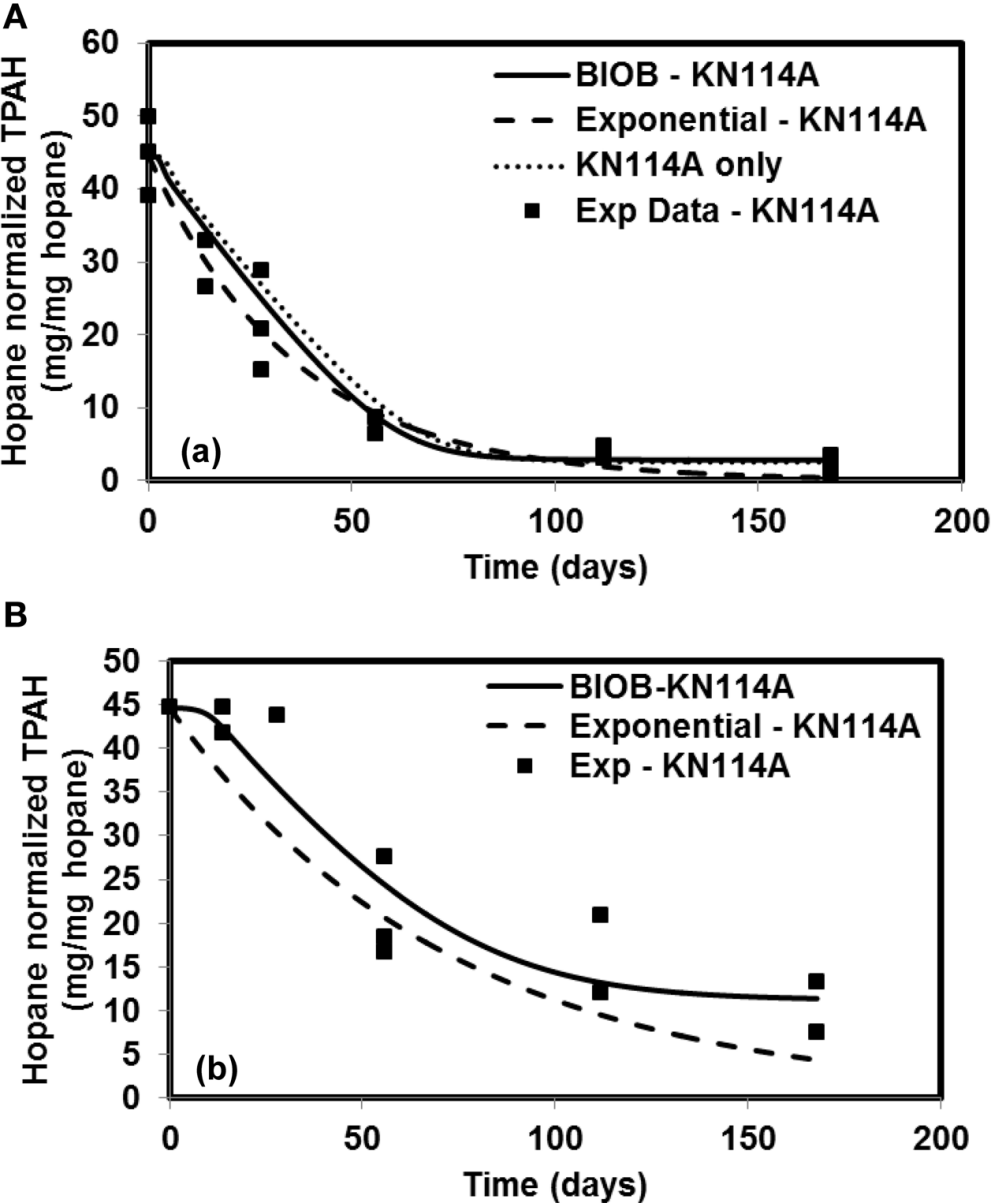
**Comparison of model results and the experimental results for Knight Island (KN114A) (A) Nutrient amended experiments (B) Natural attenuation**.

**Figure 3:**
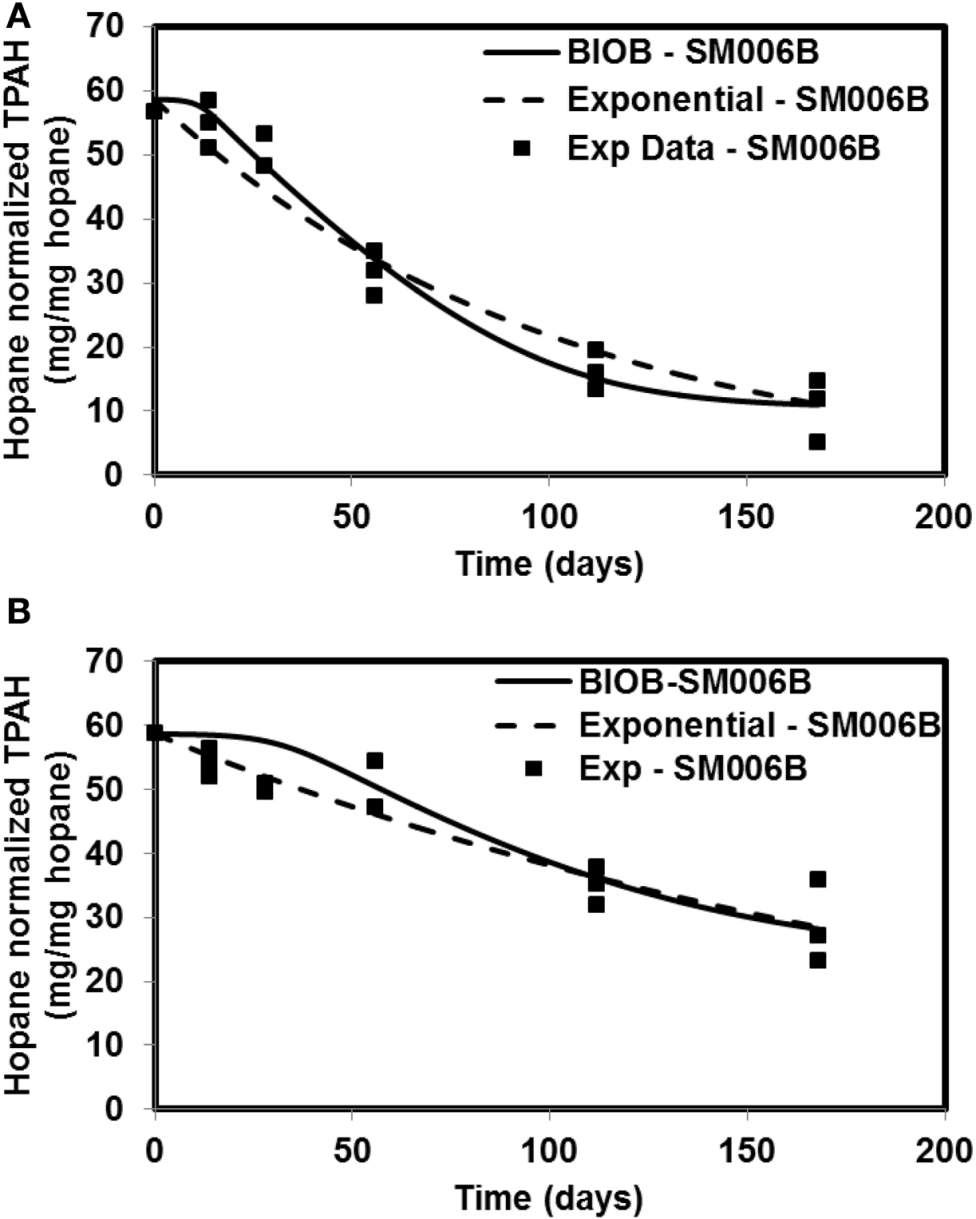
**Comparison of model results and the experimental results for Smith Island (SM006B) (A) Nutrient amended experiments (B) Natural attenuation**.

The time-varying hopane-normalized PAH concentrations for three islands (both nutrient amended experiments and natural attenuation) of EL107, KN114A, and SM006B with the parameters obtained from the GA for the nutrient amended treatments are shown in Figures 1–3, respectively. Details about the performance of parameter estimation tool with increasing generations are presented in Supplementary Material (Appendix A2). Comparisons in these Figures were made against the experimental data collected at different days and the exponential fit. The comparisons were also made against the individually estimated parameters for EL107 (Figure 1A) and KN114A (Figure 2A) locations. It can be observed from these Figures that the results from the model simulations predicted the experimental results well. It can also be observed from the Figures that the results from the combined data parameter estimates and the individual island data parameter estimates are similar.

It can be observed from Table 2 that the biomass growth rate (μ_max_) for SM006B location is four times lower compared to the other two islands. However, the values of biomass decay rate (*k*_*d*_) and half saturation constant for nitrogen consumption (*K*_*N*_) are similar for all three locations. This suggests that the biodegradation kinetics was different at the SM006B location. The differences in biodegradation kinetics could be due to the fact that the interfacial area between the oil and water may have been a limiting factor in SM006B (Geng et al., 2013). The composition of the oil could also be different as the presence of heavier PAH compounds could reduce the overall biodegradation rate. To check for the possible limitation of interfacial area, the specific surface area per unit mass was evaluated for each of the islands by using the grain size distribution obtained from the literature. The average diameter of the sediment was 4.2 mm (Li and Boufadel, 2010), 7 mm (Xia et al., 2010), and 9.4 mm (Xia and Boufadel, 2011) for Eleanor Island (EL056C), Knight Island (KN114A), and Smith Island (SM006B), respectively. The grain size distribution of Eleanor Island presented in this study is not from a same beach as was used in the Venosa et al. (2010) study. The value was used as a representative sample for this study as a qualitative analysis. The grain size distribution can be used to calculate the specific surface area per mass of sediment (*A*_*S*_) as follows (Geng et al., 2013):

(6) $$A_{S}=\frac{6}{\varphi d_{avg}\rho_{\;sediment}}$$

Where ϕ is the shape factor, *d*_*avg*_ is the average grain size, and ρ_*sediment*_ is the density of sediment.

It can be observed from Equation 6 that the specific surface area per unit mass is inversely proportional to average grain size. It was assumed that all the particles are spherical for simplicity and the value of shape factor was taken equal to 1.0. The specific surface area calculated using the above equation for EL107, KN114A, and SM006B was 0.86, 0.52, and 0.39 m^2^/kg, respectively. This indicates that the specific surface area per mass of sediment was lower in the SM006B location than the other two locations and could be one of the reasons for low interfacial area between oil and water and hence low biodegradation rates. This is reflected in the low values of growth rate (μ_max_) and maximum biomass accumulation values (as observed in Table 2) for the SM006B location.

In order to compare the goodness of fit for the exponential fit and BIOB, the objective function (WLS) was evaluated. The comparison of objective function values for BIOB and exponential fit are presented in Table 3. A lower value of the objective function indicates that the model results are closer to the experimental data. Therefore, for nutrient amended experiments, BIOB provided a better fit than the exponential fit for KN114A and SM006B datasets and the exponential fit is marginally better for EL107 dataset. For the natural attenuation experiments, BIOB provided a better fit for EL107 and KN114A whereas the exponential fit provided a marginally better fit for SM006B. BIOB's prediction for SM006B in the nutrient amended experiments is also more intuitive as there is a slight lag before the actual biodegradation begins whereas the exponential fit indicates an immediate biodegradation. This is not accurate because the biomass requires some time to grow before they are able to biodegrade the PAHs and this observation is consistent with the experimental data and hence a better fit than the exponential model as shown by the objective function values.

**Table 3:** **Comparison of the objective function values between the BIOB and Exponential models**.

	**BIOB**	**Exponential**
EL107^a^	0.3	0.15
KN114A^a^	0.28	0.97
SM006B^a^	0.014	0.06
EL107^b^	0.002	0.013
KN114A^b^	0.11	0.63
SM006B^b^	0.029	0.012

a *Nutrient amended treatment*.

b *Natural attenuation*.

*Lower value of objective function is preferred because it indicates that the model results are closer to the experimental results*.

## Sensitivity analysis

The sensitivity analyses were performed only for the nutrient amended experiments as the only parameter that was estimated in natural attenuation experiments was nutrient concentration and we present the sensitivity to nutrient concentration in a section Sensitivity to Nutrient Concentration. Based on the Equation 5, the variance of errors (σ^2^) at the optimum was 0.038 for EL107 and KN114A, and 0.0017 for SM006B for the nutrient amended experiments.

**Figure 4:**
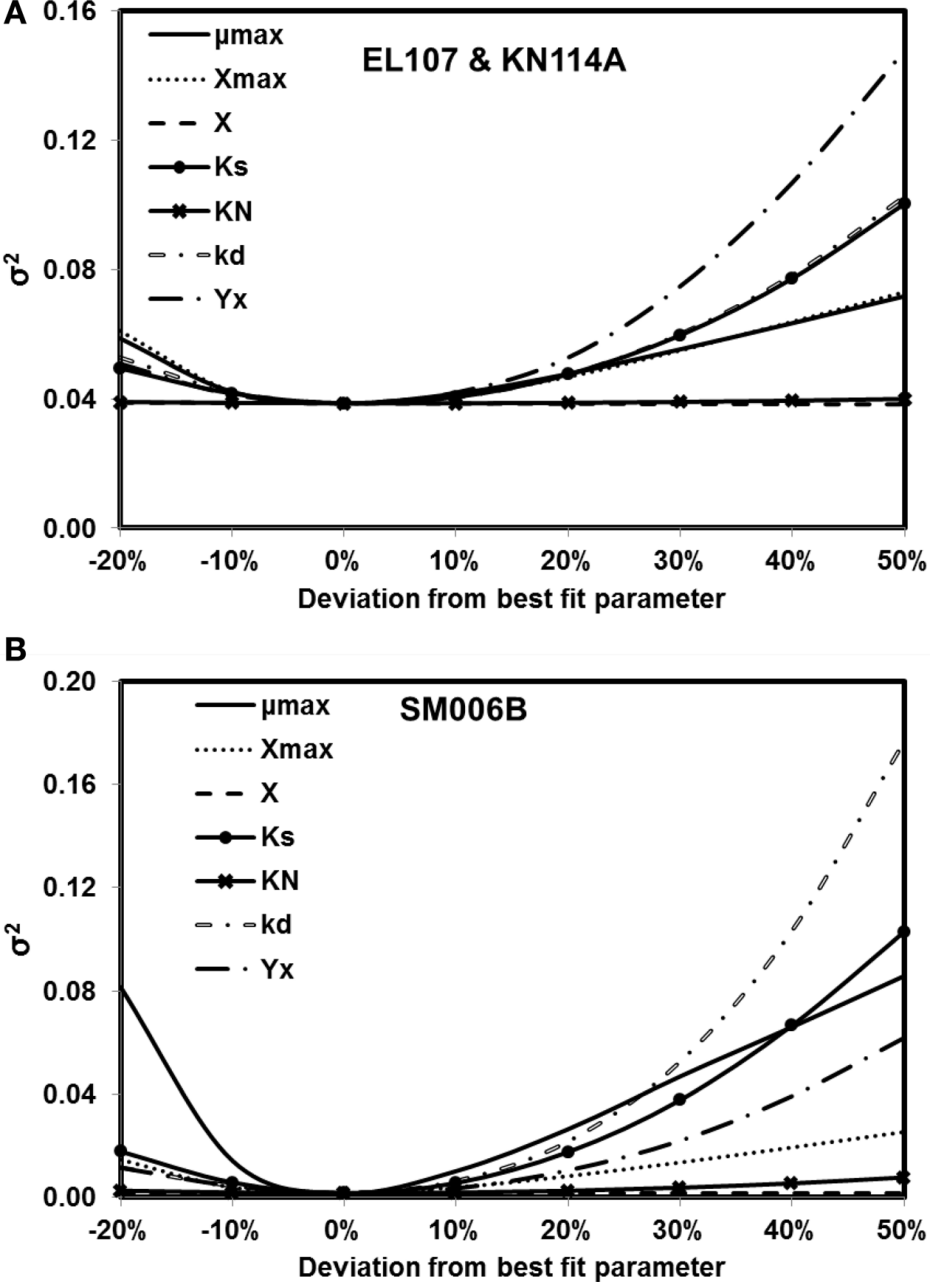
**Sensitivity analysis for kinetic parameter estimates for (A) EL107 and KN114A (B) SM006B**. Note that the minimum value of the variance of errors is at 0% deviation (i.e., estimated parameters).

The sensitivity of the parameter estimates was checked by varying each parameter between −20 and +50% of its original value while the other parameters were kept the same and the fitness was re-evaluated with the deviated parameters. Figures 4A,B show the results from analyses for EL107 and KN114A, and SM006B, respectively. It can be observed from Figure 4A (EL107 and KN114A) that the model is not sensitive to parameters *K*_*N*_ and the initial biomass concentration (*X*). The model is sensitive to yield coefficient (*Y*_*X*_), biomass decay rate (*k*_*d*_), half saturation constant (*K*_*S*_), maximum allowable biomass concentration (*X*_max_) and biomass growth rate (μ_max_). In Figure 4B (SM006B), the model is not sensitive to the initial biomass concentration (*X*), slightly sensitive to half saturation constant of nitrogen (*K*_*N*_), moderately sensitive to the parameter *X*_max_, and *Y*_*X*_, and highly sensitive to the parameters *K*_*S*_, μ_max_, and *k*_*d*_. The difference in sensitivities for the two systems as shown in Figures 4A,B indicates that the biodegradation kinetics of SM006B is indeed different from the remaining datasets. The sensitive parameters are different for each system and one needs to pay attention to these differences when employing a field scale bioremediation process. Researchers who use parameter estimation tools to estimate bioremediation parameters should pay attention to the highly sensitive parameters as small variations can change the fitness values significantly. Finally, it can be observed that the minimum value of the fitness function was obtained at 0% deviation and this shows the robustness of the search algorithm.

To quantify the findings from Figures 4A,B, the covariance matrix, *V*_*x*_, of the parameters was evaluated according to the Equation 4. To estimate the values of the Hessian matrix (*H* in Equation 5), a quadratic function can be fitted to the objective function vs. deviation from the best fit parameter (as shown in Figures 4A,B) and the second derivative of this fitted quadratic function was calculated. For the sake of simplicity, only the diagonal values of the Hessian matrix were evaluated for this study. The standard deviation values evaluated from the covariance matrix for each parameter are presented in Table 2 along with the best fit parameters. The value of standard deviation is large for some parameters like initial biomass concentration and half saturation constant for nitrogen consumption. This is due to low curvature of the objective function for these parameters and this indicates the low sensitivity to these parameters. Also the standard deviation values were quite small for the rest of the parameters and there was no overlap between the two systems except for *K*_*N*_ and *k*_*d*_ values. This further validates the hypothesis that the biodegradation kinetics of SM006B was indeed different from the other two islands.

### Sensitivity to nutrient concentration

**Figure 5:**
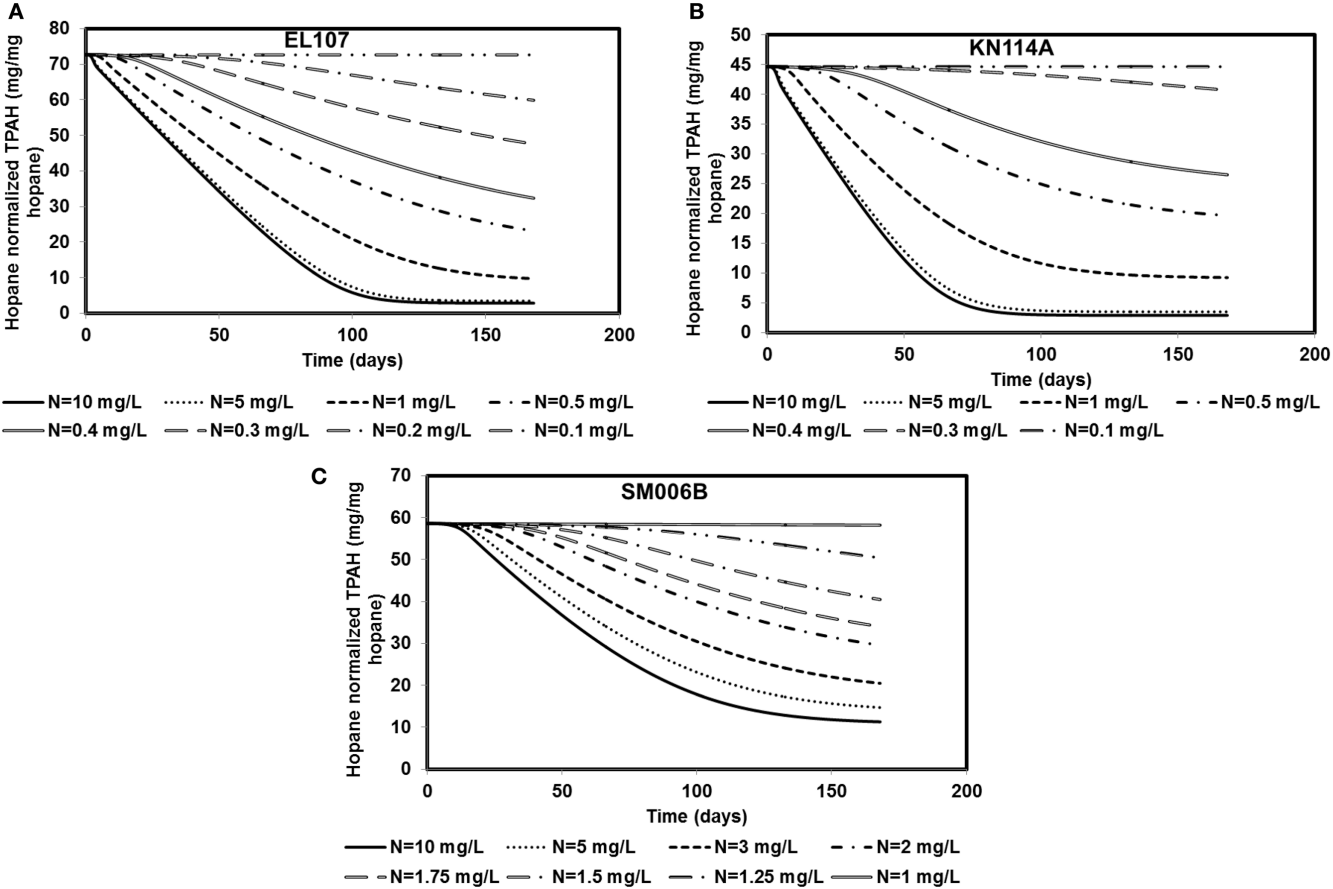
**Sensitivity of the numerical model to nutrient concentration for (A) EL107, (B) KN114A, (C) SM006B**. Note that SM006B location has slower biomass growth rate and hence it is more sensitive to nutrient concentration.

Nutrient concentration is an important parameter in biodegradation, as it has been observed by several researchers that the nitrate concentration should be about 2–10 mg/L for near maximum biodegradation rate (Atlas and Bartha, 1972; Atlas, 1981; Boufadel et al., 1999; Du et al., 1999; Wrenn et al., 2006). The nutrient concentration for the base case scenario was 10 mg/L. Simulations were performed with different nutrient concentrations between 0.1 and 10 mg/L for EL107 and KN114A locations whereas for the SM006B location, the nutrient concentration was varied between 1 and 10 mg/L. The results are presented in Figures 5A–C for EL107, KN114A, and SM006B datasets, respectively. It can be observed from the Figures that for all datasets that, as the nutrient concentration decreases, the biodegradation rate continues to decrease. Table 4 reports the predicted PAH concentrations as a percentage of the initial concentration at 168 days for various nutrient concentrations for all sites. As the nutrient concentration increases, the PAH concentration at 168 days becomes smaller. For EL107 and KN117, the concentration was affected by small increase in the nutrient concentrations for values less than 1.0 mg-N/L. Such was not the case for SM006B, where nutrient concentrations less than 1.0 mg-N/L had no effect on oil biodegradation. For this reason, the lowest nutrient value for SM006B in Table 4 was 1.0 mg-N/L, and such a value resulted in 99.2% of the initial PAH concentration remaining at 168 days. This might provide a partial explanation of the slow biodegradation of oil in the beaches of Smith Island, including SM006B.

**Table 4:** **PAH concentrations as percentage of the initial concentrations for different nutrient concentrations at *t* = 168 days**.

	**PAH concentration (mg/mg hopane)**			**PAH concentration (mg/mg hopane)**
	**EL107**	**KN114A**		**SM006B**
**Initial PAH concentration**	**72.65**	**44.68**	**Initial PAH concentration**	**58.68**
**Nutrient concentration (mg-N/L)**	**Percent of initial concentration**		**Nutrient concentration (mg-N/L)**	**Percent of initial concentration**
0.1	100	99.9	1.00	99.2
0.2	82	99.8	1.25	86
0.3	66	91	1.50	69
0.4	44	59	1.75	58
0.5	32	44	2.00	50
1.0	13	21	3.00	35
5.0	5	8	5.00	25
10.0	4	6	10.00	19

*Percentages are rounded*.

### Sensitivity to initial biomass concentration

**Figure 6:**
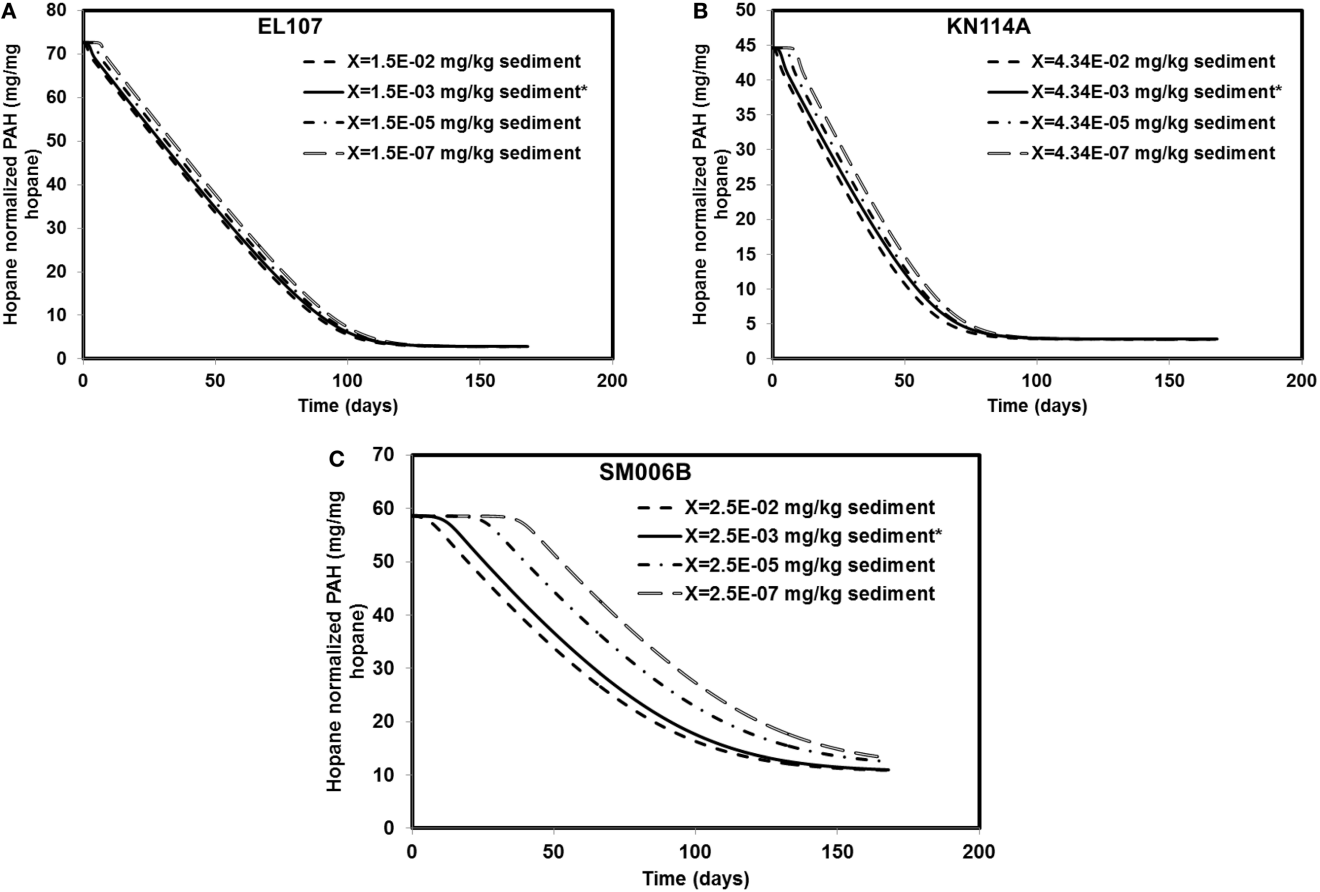
**Sensitivity of the numerical model to biomass concentration for (A) EL107, (B) KN114A, (C) SM006B**. Note that X represents the initial biomass concentration. ^*^ in the legend indicates the base case scenario.

Different scenarios were chosen in addition to the base case scenario to study the sensitivity of the system to the initial biomass concentration. The initial biomass concentrations for the base case were 1.5E-3, 4.34E-03, and 2.46E-03 mg X/kg of sediments for EL107, KN114A, and SM006B, respectively. The order of magnitude of the initial biomass concentration was increased 10-fold and decreased 100, 10,000-fold for EL107, KN114A, and SM006B datasets. The results from these simulations are presented in Figures 6A–C for EL107, KN114A, and SM006B locations, respectively. It can be observed from the Figure 6A (EL107) and Figure 6B (KN114A) that the model is not very sensitive to the initial biomass concentration after the first few days. This may be due to a high nutrient concentration and a relatively high growth rate of the bacteria in EL107 and KN114A locations. This allows the bacteria to grow rapidly and reach the maximum allowable concentration in the system. As a result, the oil biodegradation is not adversely impacted after the initial phase in EL107 and KN114A locations. The final hydrocarbon concentration after the 168 day experiment was similar for all initial biomass concentrations for both these locations. However, for the SM006B location (Figure 6C), the initial lag before biodegradation continued to increase as the initial biomass concentration was decreased. This is due to the fact that biomass growth rate is slower and hence the biomass takes longer to grow. There was a decrease in the PAH biodegradation by 14 and 20% when the biomass concentration was decreased by 100 and 10,000-fold. There was no difference in the final PAH concentration when the biomass concentration was increased 10-fold. Therefore, a low initial biomass concentration does not affect the overall biodegradation rate in EL107 and KN114A case due to its faster biomass growth rate whereas the initial biomass concentration seems to play a significant role in the SM006B case when it was decreased. This suggests that the biodegradation kinetics was not limited by the initial biomass concentration as the biodegradation happens almost immediately in the case of EL107 and KN114A (Figure 3A). This further supports the hypothesis that the interfacial area between oil and water plays a significant role in bioremediation than initial biomass concentration.

## Discussion

To compare the biodegradation rate of the polycyclic aromatic compounds (PAHs) with the biodegradation rate of individual compounds, we identified the compound with first-order decay rate similar to that of the PAHs for each location as reported by Venosa et al. (2010). This compound was designated as the representative compound which means that the rate of biodegradation of PAH is representative of the biodegradation rate of the identified compound. The first-order biodegradation rates were 0.017, 0.0294, and 0.0099 day^−1^ for nutrient amended experiments and 0.0072, 0.0138, and 0.0043 day^−1^ for natural attenuation experiments of EL107, KN114A, and SM006B, respectively. Half-life (*t*_1/2_) was evaluated from the first order decay rates (k) as follows:

(7) $$t_{1/2}=\frac{\ln2}{k}$$

Table 5 shows the names of the representative compounds and their respective half-life values for nutrient amended experiments. The first-order decay rates for the hopane-normalized individual compounds are available in the supplementary information of Venosa et al. (2010) and Equation 7 was used to evaluate the half-life values. These values are reported in Table 5 for nutrient-amended experiments. In addition to the representative compound, the slowest and fastest biodegradable compounds are also identified for each island. Table 5 also shows the fastest and slowest biodegradable compounds for each island along with their half-life values. Table 6 provides the names and half-life values of the representative compounds as well the fastest and slowest compound for the natural attenuation experiments.

**Table 5:** **Comparison of biodegradation rates for nutrient-amended experiments**.

**Island**	**PAH half life**	**Representative compound (Venosa et al., 2010)**	**Half life (Venosa et al., 2010)**	**Fastest compound**	**Half life (Venosa et al., 2010)**	**Slowest compound**	**Half life (Venosa et al., 2010)**
EL107	40.8	C2-Fluorene	38.9	Phenanthrene	4.3	C4-Chrysene	888.7
KN114A	24.4	NBT	22.9	C1-Fluorene	11.9	C4-Chrysene	330.1
SM006B	70.0	C2-Nap/C3-DBT	71.5	DBT	24.8	C4-Chrysene	462.1

*C2-Nap represents C2-Naphthalenes, NBT—Naphthobenzo-thiophenes, and DBT represents Dibenzothiophene. All units for half-life values are in days*.

**Table 6:** **Comparison of biodegradation rates for natural attenuation experiments**.

**Island**	**PAH half life (Venosa et al., 2010)**	**Representative compound (Venosa et al., 2010)**	**Half life (Venosa et al., 2010)**	**Fastest compound**	**Half life (Venosa et al., 2010)**	**Slowest compound**	**Half life (Venosa et al., 2010)**
EL107	96.3	C1-Naphthalene	97.6	Phenanthrene	10.3	C4-Chrysene	6931.5
KN114A	50.2	NBT	51.0	C4-Naphthalene	19.6	C3-NBT	1980.4
SM006B	161.2	C2-DBT	165.0	DBT	23.0	C4-Chrysene	1386.3

*NBT—Naphthobenzo-thiophenes and DBT represents Dibenzothiophene. All units for half-life values are in days*.

**Figure 7:**
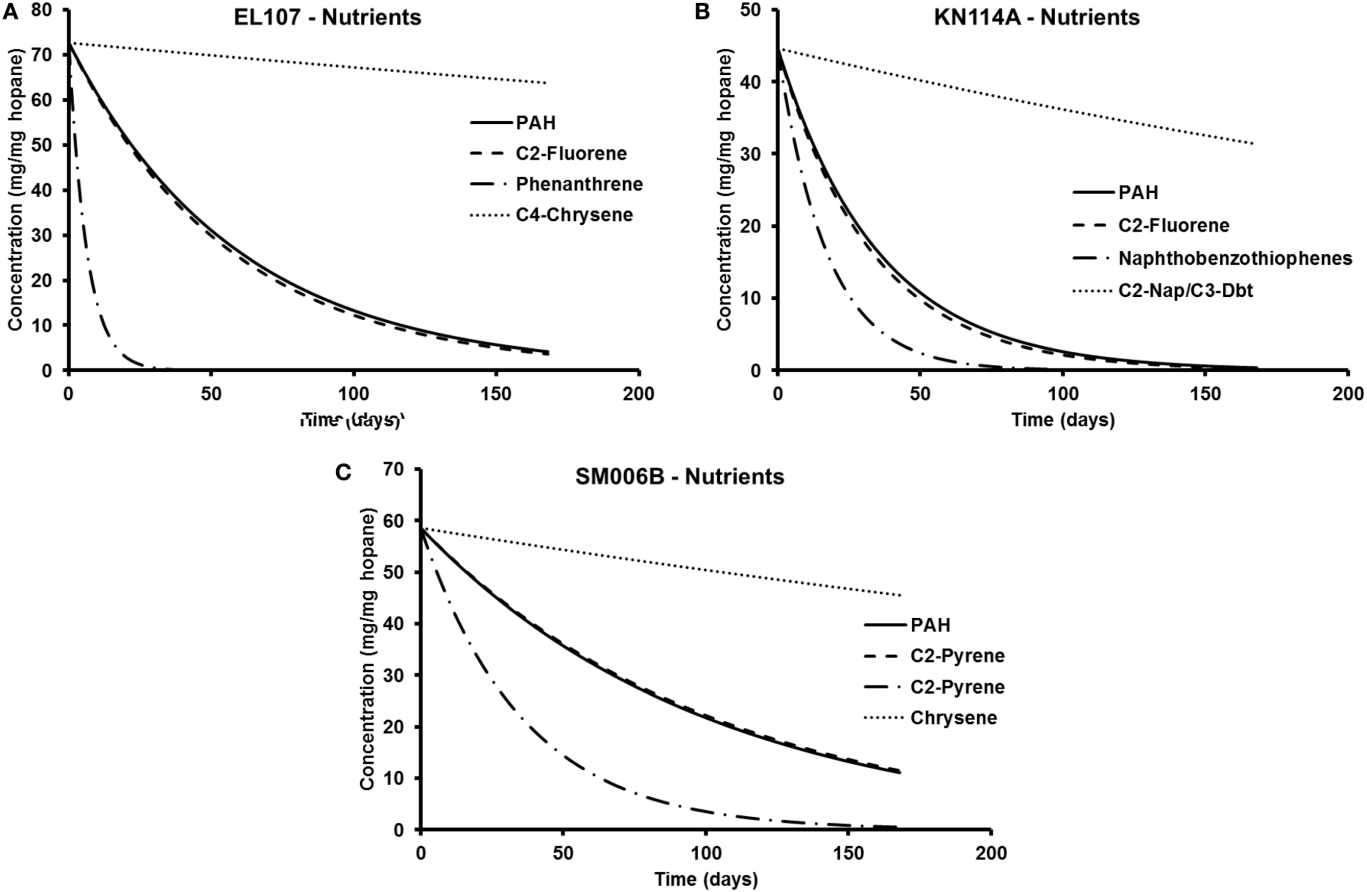
**Comparison of the temporal variation for different compounds in nutrient-amended experiments for (A) EL107, (B) KN114A, and (C) SM006B**.

**Figure 8:**
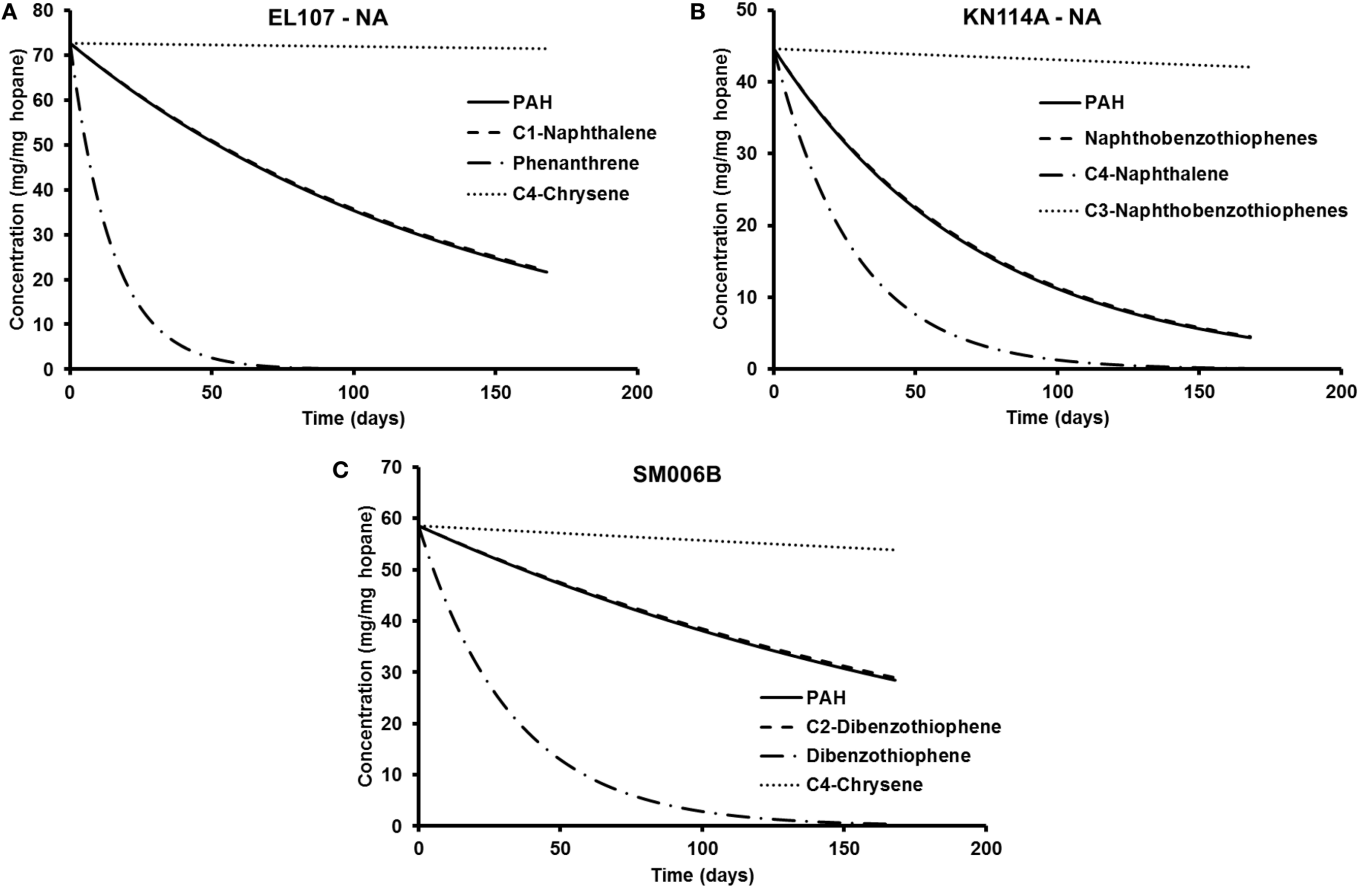
**Comparison of the temporal variation for different compounds in natural attenuation experiments for (A) EL107, (B) KN114A, and (C) SM006B**.

The parameters in Tables 5, 6 are used to plot the temporal variation of the concentration of hydrocarbon for each island. It was assumed that the respective compounds have the same concentration as the initial PAH concentration at that island to allow for easy comparison. The results from the simulations are shown in Figures 7A–C for EL107, KN114A, and SM006B, respectively, for the nutrients amended experiments and Figures 8A–C show simulation results for natural attenuation experiments for EL107, KN114A, and SM006B, respectively. It can be observed from the Figures that the biodegradation rates vary vastly between different compounds.

We have also compared the number of compounds with biodegradation rate higher and lower than the representative compound for that island. For the nutrient-amended experiments, there were 10 compounds biodegrading at higher rate than the representative compound for all islands whereas there were 17, 11, and 13 compounds biodegrading at lower rate than the representative compound for EL107, KN114A, and SM006B Islands, respectively. Similarly, for the natural attenuation experiments there were 11, 9, and 9 compounds biodegrading at higher rate than the representative compound for EL107, KN114A, and SM006B, respectively, whereas there were 16, 12, and 15 compounds biodegrading at lower rate than the representative compound for EL107, KN114A, and SM006B Islands, respectively.

The weighted rates at each site were evaluated by multiplying the initial concentration of the compound with its rate and this summation was divided by the concentration of the PAH at the site. These weighted rates were used to compute the half-life using Equation 7 and comparison between the half-life computed using exponential curve fitted data and weighted half-lives are shown in Table 7. It can be observed from the Table that the weighted half-lives for each site are lower than the half-lives computed from the exponential curve fitted data. It should also be noted that BIOB is capable of estimating the biodegradation of individual hydrocarbons where information about these compounds such as the concentrations of the hydrocarbon and the microbes capable of biodegrading that hydrocarbon are available. In Equations 1 and 2, the term *S* will represent the concentration of the hydrocarbon instead of the PAH concentration if we use BIOB to estimate the biodegradation of individual compounds.

**Table 7:** **Comparison of the half-lives between the weighted and fitted decay rates**.

	**Weight half-life (days)**	**Fitted half-life (days)**
EL107—Nutrients	25.26	40.77
KN114A—Nutrients	20.06	24.41
SM006B—Nutrients	60.93	70.01
EL107—NA	57.81	96.27
KN114A—NA	36.64	50.23
SM006B—NA	140.26	161.20

*NA represents natural attenuation*.

**Figure 9:**
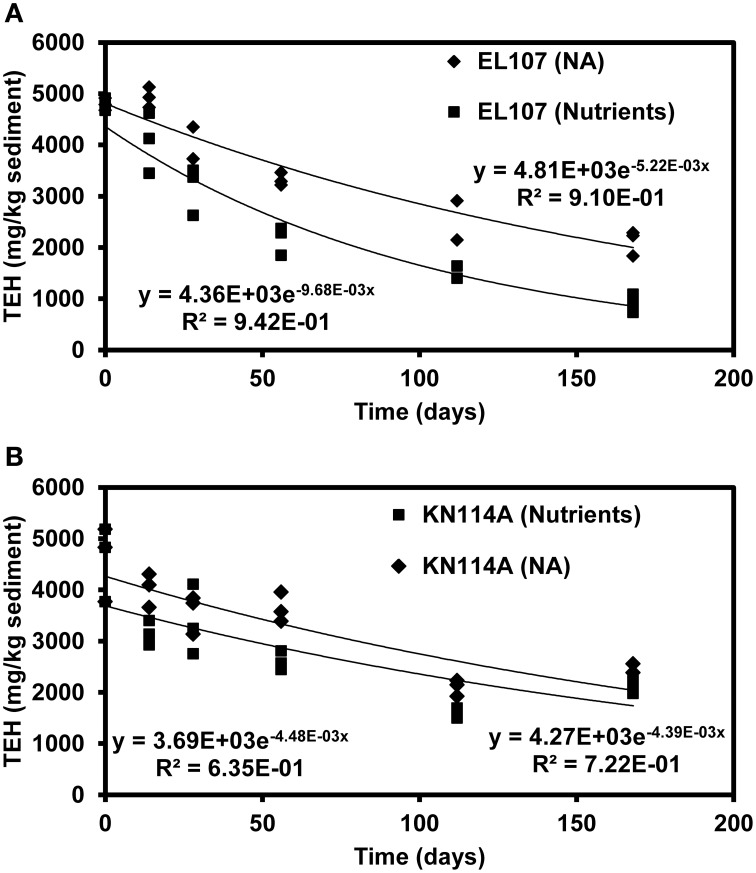
**Decrease of TEH (total extractable hydrocarbon) as function of time in (A) the EL107 and (B) KN114A microcosms along with fitted exponential decay curves**. Diamond symbols = Natural attenuation, and square symbols = Nutrient amended.

**Figure 10:**
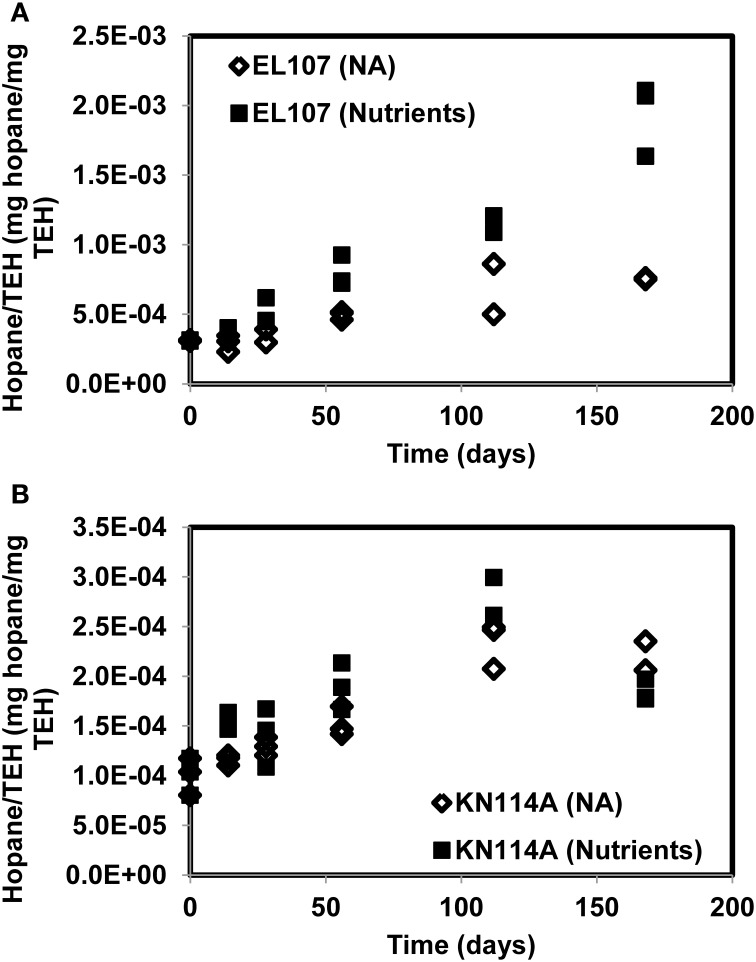
**Hopane concentration normalized by the concentration of TEH (total extractable hydrocarbon) for (A) the EL107 and (B) the KN114A microcosms**. The overall increase with time reflects biodegradation.

Figures 9A, B report the concentration of total extractable hydrocarbon (TEH) as function of time in the microcosms of EL107 and KN114A, respectively. Simple exponential decays were fitted to illustrate the decreasing behavior. The Natural Attenuation data for EL107 (Figure 9) show a lag in the decrease of TEH with time, similar to that observed for the hopane-normalized PAH concentration for EL107 (Figure 2B), which suggests that the lag is due to biotic factors, namely the initial phase of microbial growth (or microbial acclimation period). For KN114A, the decrease is more or less immediate for both data sets (Figure 10), but a sudden drop in the TEH concentration occurs at 120 days for all microcosms. We have considered and ruled out any possibility for this decrease, which occurred for both Natural attenuation and nutrient amended microcosms. We believe these data points do not represent correct results, but they don't invalidate the overall trend of decrease with time.

Figure 10 reports the concentration of hopane normalized by the total mass of TEH for EL107 and KN114A. The increase with time reflects the biodegradation of compounds less resistant to biodegradation than hopane. For KN114A, the decrease between 120 and 180 days reflect the low TEH values at 112 days (Figure 9B). The sudden decrease cannot be due to the biodegradation of hopane, because although hopane does biodegrade, its time scale of biodegradation is longer than a few weeks, more on the order of years (Atlas and Bragg, 2007).

**Figure 11:**
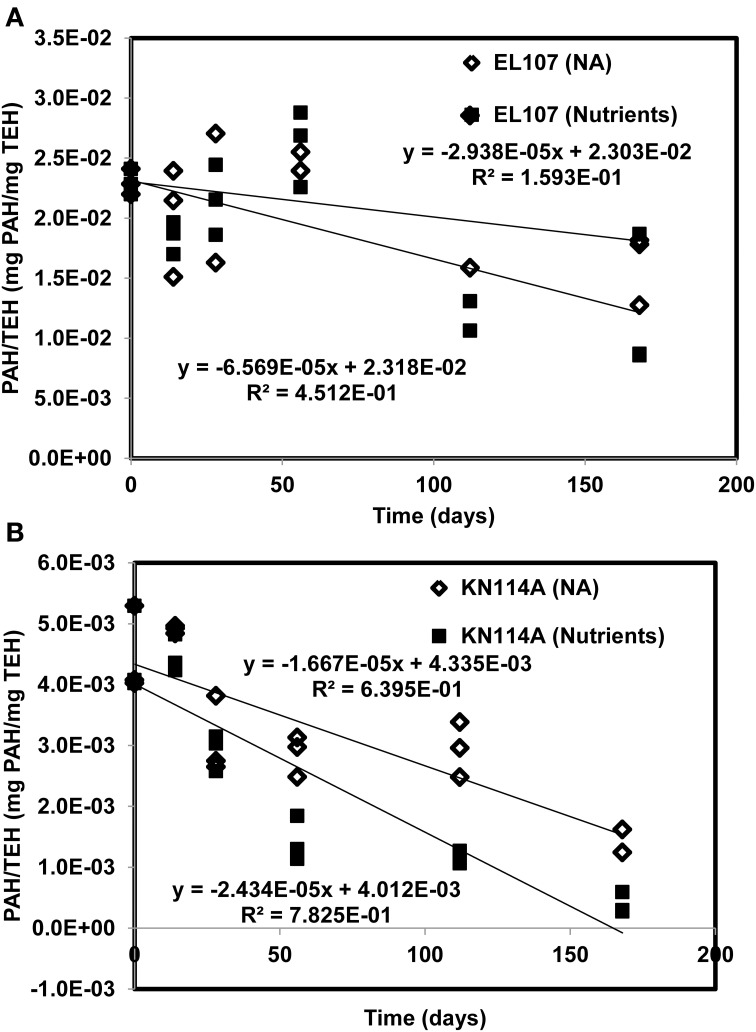
**PAH concentration normalized by the concentration of total extractable hydrocarbon (TEH) as function of time for (A) EL107 and (B) KN114A**. Fitted straight lines were obtained to determine if there is a correlation between the biodegradation of PAH and that of TEH.

Figures 11A, B report the PAH concentration normalized by the concentration of TEH as function of time for (A) EL107 and (B) KN114A. Fitted straight lines were obtained to determine if there is a correlation between the biodegradation of PAH and that of TEH. For EL107, a low *R*^2^ was noted along with a small slope, which suggests that the biodegradation of PAH is not directly related to the biodegradation of TEH. We think this has to do also with the variability of the TEH at 112 days. However, the high *R*^2^ for KN114A suggests a strong linear relation between the biodegradation of PAH and that of TEH. At face value, Figure 11 suggests that tracking the biodegradation of PAHs is not sufficient to infer the biodegradation of the rest of the compounds (i.e., TEH).

It should also be noted that the seawater that was renewed to the microcosms was recirculated to the reservoir that supplies the seawater to the microcosms. Therefore, in case of oil leeching, it would probably be replenished to the microcosms when the seawater is renewed. The leaching of oil is also not mentioned in Venosa et al. (2010) and the hopane concentration remains fairly constant throughout the length of the experiment which means the removal of oil due to physical processes was limited. Therefore, we did not account for leaching in the model.

## Conclusions

In this study, the biodegradation model BIOB was used to simulate the experimental data from nutrient amended and the natural attenuation treatments from Venosa et al. (2010) study. The kinetic parameters required for the BIOB were estimated using a parameter estimation tool based on GA. It was observed from the estimated parameters that the SM006B location had relatively slower biodegradation rate compared to the other two locations. Grain size analysis from each site revealed that the specific surface area per unit mass of sediment was considerably lower at SM006B, which suggests that the surface area of sediments is a key control parameter for microbial growth in sediments. Subsequently, the best-fit parameters obtained from the nutrient amended experiments were used to estimate the nutrient concentration for the natural attenuation experiments. The estimated parameters were able to predict the experimental results well for all the datasets. In comparison with the exponential decay model, the results from BIOB showed better goodness of fit for KN114A and SM006B datasets while the fit for EL107 dataset for nutrient amended experiments was relatively similar for both exponential decay and BIOB. For natural attenuation treatment, the exponential fit provided a better fit for the SM006B dataset whereas BIOB provided a better fit for EL107 and KN114A datasets. In particular, BIOB was able to capture the initial slow biodegradation due to the lag phase in microbial growth. In addition, the exponential fit does not account for the limitation of nutrients and has no predictive capabilities whereas the parameters estimated for BIOB are still valid at different nutrient concentrations. Therefore, BIOB can be used to predict the bioremediation scenarios at the site.

It was observed from our sensitivity analyses that the initial biomass concentration and the half saturation coefficient of nitrate were not sensitive parameters for EL107 and KN114A locations whereas the biomass growth rate, biomass decay rate, yield coefficient, half-saturation constant for hopane-normalized PAH, maximum allowable biomass were the most sensitive parameters. For the SM006B location, the system was highly sensitive to biomass decay rate, biomass growth rate, half saturation constant for hopane-normalized PAH, yield coefficient and slightly sensitive to maximum allowable biomass concentration and half-saturation constant for nitrogen, and insensitive to small changes in initial biomass concentration. In addition, when the sensitivity analyses were performed on the nutrient concentration, SM006B location was more sensitive to small changes than EL107 and KN114A locations due to the slow biomass growth rate. Even though the nutrient concentration was a sensitive parameter, there was no significant difference in concentration trends for the nutrient concentrations of 5 and 10 mg of N/L in EL107 and KN114A locations. Also, the initial biomass concentration had much lower effects on the final PAH concentration after 168 days in EL107 and KN114A locations than in the SM006B location. The high nutrient concentration allowed for a rapid biomass growth and thus, the PAH biodegradation was not substantially affected after the initial few days in the EL107 and KN114A location whereas in the SM006B location, when the initial biomass concentration was decreased by several orders of magnitude, the biomass required several days before the biomass concentration was sufficient enough to cause hydrocarbon decay. Therefore, it can be concluded that the initial biomass concentration affects oil biodegradation significantly during the initial phase of the biodegradation process. Comparisons were also made to study the biodegradation decay rates of individual compounds against the decay rate of the PAH. It was observed that the weighted average of the individual decay rates was greater than the decay rate of the PAH. In conclusion, we determine that the Monod kinetic model, BIOB is a useful tool that can be used to predict the extent of biodegradation in laboratory scale experiments and in marine environments such as beaches where the oil is entrapped within sediments with no significant transport or dissolution.

### Conflict of interest statement

The authors declare that the research was conducted in the absence of any commercial or financial relationships that could be construed as a potential conflict of interest.
