# Supplementary material for: Evaluation of the biodegradation of Alaska North Slope oil in microcosms using the biodegradation model BIOB
Source: Front Microbiol. 2014 May 14;5:212. doi: 10.3389/fmicb.2014.00212 (PMC4030209; doi:10.3389/fmicb.2014.00212)
Supplement: Supplementary file 1 [file DataSheet1.PDF]

APPENDIX A1

The numerical model, BIOB, uses Monod kinetics to simulate the growth of bacteria in the presence of nutrients and the decay of the hydrocarbon attached to the sediment. The decay of hydrocarbon can be mathematically expressed as follows (Geng *et al.*, 2014):

$$\frac{dS}{dt} = -\frac{\mu}{Y_X} X + \frac{S}{h}\frac{dh}{dt} \tag{A1}$$

Where S is the concentration of the substrate (oil) (mg S/kg sand), X is the concentration of the biomass (mg X/kg sand), Y_x is the biomass yield coefficient for growth on the hydrocarbon (mg X/mg S), h is concentration of hopane (biomarker). The term $\frac{dh}{dt}$ accounts for the removal of hopane due to physical processes and μ is the growth rate of the biomass (day^{-1}) given by (Geng *et al.*, 2014):

$$\mu = \mu_{\max}\left(1 - \frac{X}{X_{\max}}\right)\frac{S}{K_S + S}\frac{N}{K_N + N} \tag{A2}$$

Where μ_{max} is the maximum growth rate (day-^{1}), X_{max} is the maximum allowable microbial concentration (mg X/kg sand), K_s is the half saturation concentration of hydrocarbon (mg S/kg sand), N is the nitrogen-based nutrient concentration (nitrate+nitrite+ammonia) (mg-N/L of pore water), and K_N is the half-saturation concentration for nitrogen consumption (mg-N/L of pore water). The term $\left(1 - \frac{X}{X_{\max}}\right)$ was introduced by (Geng *et al.*, 2014) to account for the decrease in biomass accumulation when the microbial concentration approaches its maximum value. The biomass growth can be expressed mathematically expressed as follows (Geng *et al.*, 2014):

$$\frac{dX}{dt} = \left(\mu - k_d\right) X \tag{A3}$$

Where k_d is the endogenous biomass decay rate (day^{-1}).

Oxygen consumption was assumed to depend on the rate of substrate degradation, the decay rate of the degradable fraction of the biomass, and the total amount of biomass formed. In essence, oxygen consumption was modeled by performing a mass balance on electrons, where electrons released by oxidation of substrate and decaying biomass are consumed by reduction (Geng *et al.*, 2014).

$$\frac{dO_2}{dt} = Y_{OS}\left(\frac{\rho}{\theta}\right)\frac{dS}{dt} + Y_{OX}\left(\mu - k_d\right) X \tag{A4}$$

Where Y_{OS} (mg O_2/mg S) is the stoichiometric coefficient for oxygen consumption based on complete mineralization of substrate (Eq. 1), ρ is the bulk density of the sand, θ is the porosity of the porous media and Y_{OX} (mg O_2/mg X) is the stoichiometric coefficient for oxygen consumption during the complete mineralization of biomass.

Similarly, the CO_2 production (expressed as mg of C) is assumed to depend on the substrate consumption rate, the gross rate of biomass growth and the biomass decay rate. Carbon dioxide production was modeled using a mass balance on carbon, assuming that carbon is released by oxidation of substrate and decaying biomass and is consumed by synthesis of new biomass with the remainder ending in CO_2 (Geng *et al.*, 2014):

$$\frac{dC}{dt} = Y_{CS}\left(\frac{\rho}{\theta}\right)\frac{dS}{dt} + Y_{CX}\left(\mu - k_d\right) X \tag{A5}$$

where Y_{CS} (mg C/mg S) is the stoichiometric coefficient of carbon dioxide production from substrate and Y_{CX} (mg C/mg X) is the amount of CO_2 produced by complete mineralization of biomass.

Nitrate consumption reflected the interaction between biomass growth and decay as follows:

$$\frac{dN}{dt} = Y_N \left(\frac{\rho}{\theta} \right) \left(\mu - k_d \right) X \tag{A6}$$

Where Y_N (mg N/mg X) is the stoichiometric coefficient of N consumption from substrate.

APPENDIX-A2

The variation of fitness for the nutrient amended experiments in EL107 and KN114A, and SM006B locations is shown in Figure A1(a) and A1(b). It can be observed from the Figure that the GA reaches its best fitness of 0.58 after 122 generations for KN114A and EL107 datasets whereas for SM006B, the best fitness was 0.014 after 221 generations. There was no further change in the solutions with increasing generations. This means that the GA has reached the global minimum for the given datasets.

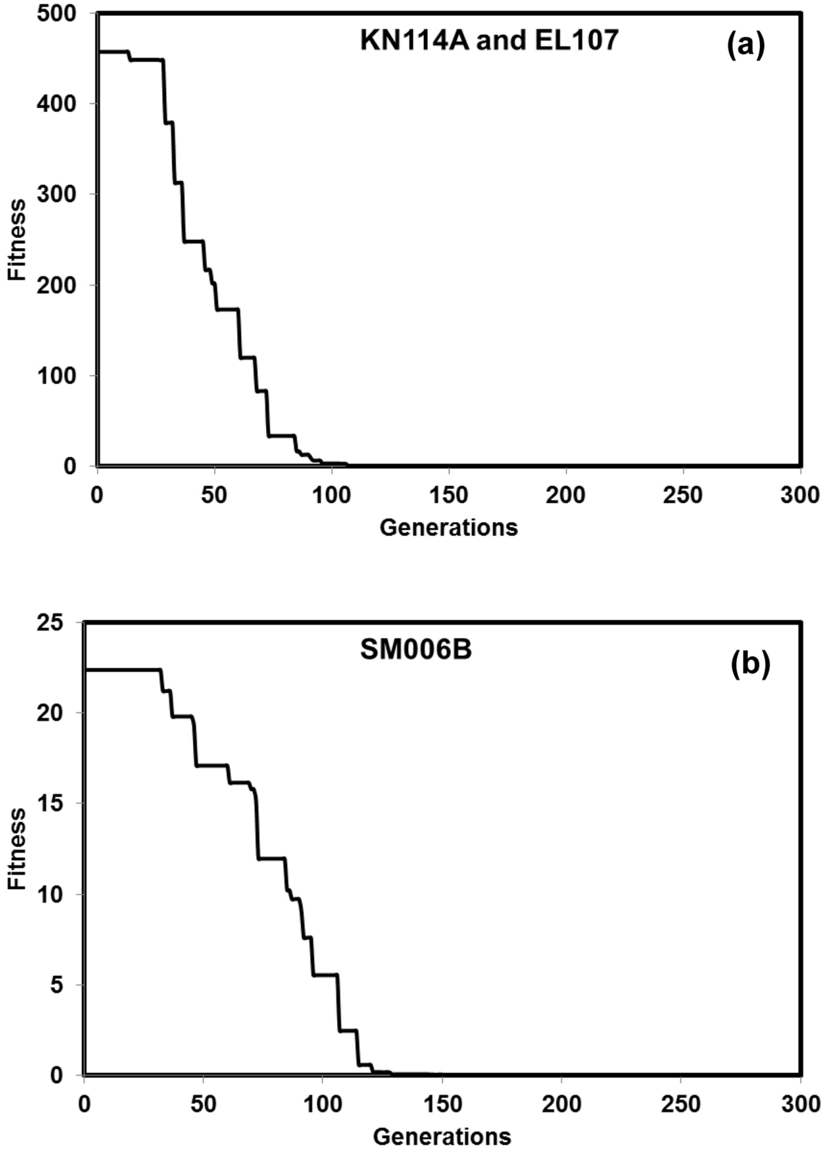

Figure A1: Variation of fitness (objective function) with the generations for the Genetic Algorithm (GA) for a) EL107 and KN114A b) SM006B
